# Effectiveness of Using Nucleic Acid Amplification Test to Screen Blood Donors for Hepatitis B, Hepatitis C, and HIV: A Tertiary Care Hospital Experience From Pakistan

**DOI:** 10.7759/cureus.34216

**Published:** 2023-01-25

**Authors:** Syeda M Ali, Naila Raza, Muhammad Irfan, Mahnoor F Mohammad, Fatima H Kazmi, Zainab Fatima

**Affiliations:** 1 Hematology and Oncology, Liaquat National Hospital and Medical College, Karachi, PAK; 2 Hematology, Liaquat National Hospital and Medical College, Karachi, PAK

**Keywords:** hiv, hcv, hbv, transfusion-transmitted infections, nucleic acid amplification test

## Abstract

Background

Ensuring blood safety is the primary goal of transfusion medicine. Despite extensive serological tests and strict safety measures, the risk of transfusion-transmitted infections (TTIs) still exists. As applied to blood screening, Nucleic Acid Amplification Test (NAT) offers much higher sensitivity for detecting viral infections. It is, however, currently available to a handful of centers due to the high cost. This study aims to establish the Effectiveness of NAT by assessing the NAT yield and residual risk of transmission of Hepatitis B virus (HBV), hepatitis C virus (HCV) and HIV with and without NAT testing.

Material and method

This prospective cross-sectional study recruited blood donors from January 2020 to November 2022. All donors underwent routine serologic screening. Only serologically negative donors were tested for HBV, HCV, and HIV by NAT. The NAT yield and residual risk (RR) per million donors were computed for viral infections in seronegative blood donors and calculated using the incidence/window period model.

Result

A total of 59708 donors were included during the study period. The overall prevalence of TTI's were: For HCV 1.7% (n = 1018), HBV 1.5% (n = 918), HIV 0.07% (n = 47), Syphilis 1.2% (n = 758) and malaria 0.3% (n = 218). Out of 57759 seronegative donors, thirty-four NAT-reactive samples were identified, with 3 cases of HCV, 31 cases of HBV, and Nil HIV cases. NAT yield of HBV was 1 in 1863 with an RR of 8.6 per million, followed by HCV with a NAT yield of 1 in 19253 and RR of 0.8 per million donations. NAT testing reduced RR for HBV by 48.9% and HCV by 94.5%.

Conclusion

Our study showed that NAT detected 34 out of 57759 cases initially missed by serological tests. The study suggests that the parallel use of serology and NAT screening of donated blood would be beneficial.

## Introduction

Every year millions of people around the globe donate and receive blood or its components. Transfusion medicine practice aims to ensure safety both for donors and recipients of blood. An integral part of this safety chain includes: the donor's detailed medical history, fulfilling strict donor deferral criteria, proper cleaning of the phlebotomy site to prevent the risk of bacterial contamination, and extensive laboratory testing of donated blood to minimize the risk of transfusion-transmitted infections (TTIs) to as low as zero level.

Despite pre-transfusion testing and strict safety measures, a "Zero-risk" blood supply has not been achieved. The risk of TTIs remains with the viruses having a long incubation period or causing subclinical infections like Hepatitis B (HBV), hepatitis C virus (HCV), and HIV. Implementing safe blood transfusion practices is quite challenging for countries like Pakistan, with limited resources and a high prevalence of HBV and HCV. Pakistan has the second highest global burden of HCV, with 5% population infected [1]. Similarly, the prevalence of HBV is 1.98% in the general population [2]. Moreover, the World Health Organization Eastern Mediterranean Region (WHO EMRO) reported a recent outbreak of HIV infection in the Larkana district, located in the northwestern part of Sindh province of Pakistan, which showed 751 HIV-positive cases of 26041 people screened [3].

The most robust method for screening blood donations are Enzyme immunoassays (EIAs) and Chemiluminescent immunoassays (CLIAs). Both tests detect the antigen-antibody reaction. However, a time lag exists between exposure to infection and the formation of antibodies against the virus, and this period is called the window period (WP) [4]. During this time, the infection in donated blood remains undetected by the EIA/CLIA testing. These undetected or occult infections during WP are the major cause of TTIs. Nucleic Acid Amplification Test (NAT) is a technique in which specific viral nucleic acid, either DNA or RNA, is targeted and amplified, which enables the detection of extremely low levels of virus in WP. Thus, NAT offers much higher sensitivity for detecting viral infections in blood donors.

NAT was introduced in the United States of America and Europe in the mid to late 1990s for screening donated blood [5]. It was considered that developing countries with a high prevalence of TTIs would benefit most from the introduction of NAT for screening blood donors. In Pakistan, NAT was launched almost 10 years ago to screen donated blood, but due to higher costs, only a few blood banks in the country have adopted NAT for blood donor screening. As a result, a small number of local studies are available supporting the efficacy of NAT [6-9].

In Liaquat National Hospital, Karachi, NAT has been used to screen seronegative blood donors since 2014. We aim to study the efficacy of NAT for HCV, HBV, and HIV in seronegative blood donors and focus on the residual risk of disease transmission with and without NAT testing.

## Materials and methods

Study setting and design

It was a prospective cross-sectional (validation) study conducted at Liaquat National Hospital, Karachi. It is a big tertiary care teaching hospital with a fully developed and equipped blood bank. Most blood donors who visited the blood bank were noncommercial replacement donors. Donated blood underwent screening by serological tests, complemented by NAT testing.

Data collection

For the sake of this study, we started collecting data from January 1, 2020, till November 30, 2022. We prospectively selected 59708 first-time donors with ages between 18-55 years. The algorithm of blood donation includes initial screening using an in-house developed questionnaire on general health conditions to reject donors who are either positive for TTIs in the past or at a high risk of having these infections. Simultaneously, CBC was done on Nihon Kohden Cell Counter (MEK-6410K) to rule out anemia. Every donor had to sign written informed consent before donation. Samples for screening tests were collected in two different tubes. Serum separator tube used for serologic screening of HbsAg, anti-HCV, HIV Ag-Ab assay, and syphilis Ab. Sample collected in EDTA tube used for NAT testing. Rapid tests were performed for malarial parasites on a commercially prepared kit. The donated blood units were quarantined until all results of screening tests to detect HIV, HCV, HBV, syphilis, and malaria were available.

Screening tests

Chemiluminescence Assay (CLIA)

Serum samples from all the donors were screened on an automated immunoassay analyzer (Architect i2000, Abbott Diagnostics, Abbott Park, IL) for HBsAg, anti-HCV, HIV Ag-Ab, and syphilis TP antibodies. The ICT method was used for the detection of malarial parasites. Donated blood that reacted positively was discarded, and the donor was informed. 

NAT Testing

Donors with negative screening for HBsAg, Anti HCV, and HIV Ag-Ab were sent to screening by NAT in the molecular pathology department of the hospital. NAT was done on a fully automated Roche Cobas 6800/8800 system using a multiplex polymerase chain reaction kit (Cobas MPX test). All the blood products of the donors were released after confirmation as non-reactive. NAT was done in individual donation (ID) format, meaning every sample was tested separately.

Window period (WP), Incident rate (IR), and Residual risk (RR)

The estimation of IR and RR is based on the NAT yield /WP model [10]. Window period estimation of HIV, HBV, and HCV are derived from literature [10,11]. The formula for calculating NAT yield and Residual risk [12] is given in Table 1. ID-NAT WP (T1) is the period between infectivity and ID-NAT detectability. These were taken for HBV, HCV, and HIV at 10.3, 1.3, and 2.9 days, respectively [4]. The period between ID-NAT and CLIA detectability (T2) for HBV, HCV, and HIV was taken as 23.6, 32.6, and 8.6 days respectively (Package insert - Cobas MPX - FDA) [12]. Serologically non-reactive cases who were NAT reactive were considered incident cases.

**Table 1:** Formulas for calculation of RR RR- Residual risk; NAT: Nucleic Acid Amplification Test;

FORMULAS:
NAT Yield = (Incident cases/No. donors screened by NAT) X 100
Serologic prevalence per 10^5^ donation= (No. of serologically positive donors/ Total no. of donors)X 10^5^
Yield rate per 10^6^ donors = (NAT yield/ No. donors screened by NAT)X10^6^
Residual risk per million donors = yield rate X Time ratio (T1/T2)

Statistical analyses

Data was taken from the blood bank screening records, entered on a Microsoft Excel sheet, imported on IBM SPSS version 22, and then analyzed. Prevalence of HBV, HCV, HIV, Syphilis, and malaria was expressed in percentages. Incident cases on NAT yield of HBV, HCV and HIV are also expressed in percentages. Pearson Chi-squire (χ2) test or Fisher's exact test was used to evaluating the relationship between categorical variables. A p-value < 0.05 was considered statistically significant.

## Results

Socio-demographic characteristics of donors

During the study period, 59,708 blood donors donated blood at Liaquat National Hospital, Karachi. Among those donors, 59,660 (99.9%) were males, and 48 (0.08%) were females. Most donors were replacement donors, i.e., 58806 (98%), while a small number of donors, i.e., 902 (2%), donated blood voluntarily. The mean age of blood donors was 36 years, ranging from 18 to 55 years. Almost half of them, i.e., 52%, were between 18-30 years, whereas 29% were between 31-40 years and 19% were more than 40 years of age.

Serologic screening

Fifty-nine thousand seven hundred-eight blood units underwent serologic testing for HBV, HCV, and HIV, and 1949 units were found to be reactive. 887 (1.5%) units were found to be positive for HbsAg, 1015 (1.7%) were found to be positive for anti-HCV, and 47 (0.07%) units were HIV Ag/Ab positive.

NAT yield

Of the 57759 seronegative blood donors, 31 were found to be reactive for HBV, and 3 were HCV reactive. No HIV case was detected. Overall NAT yield was 1 in 1699 donors, with 1 in 1863 donors for HBV and 1 in 19253 donors for HCV (Table 2).

**Table 2:** NAT yield

Virus	Seronegative donors	No. of incident cases	NAT yield (%)	Yield rate
Hepatitis B	57759	31	0.05	1 in 1863
Hepatitis C	57759	3	0.005	1 in 19253
Total	57759	34	0.05	1 in 1699

Prevalence of TTIs (Serology + NAT+ICT)

The study showed that overall prevalence for HBV, HCV, HIV, malaria and syphilis were found to be 1.5% (n=918), 1.7% (n=1018), 0.07% (n=47), 0.3% (n=218), 1.2% (n=758) respectively with HCV infection having the highest prevalence followed by HBV (Table 3).

**Table 3:** Reactive cases among blood donors (Serology+NAT+ICT) *ICT method used n/a=not available NAT: Nucleic Acid Amplification Test; TTIs: transfusion-transmitted infections; ICT: Immunochromatographic Test.

TTI’s	Total donors (n)	Reactive (Serology)	NAT reactive	Total reactive (%)
HCV	59708	1015	3	1018(1.7)
HBV	59708	887	31	918(1.5)
HIV	59708	47	0	47(0.07)
Syphilis	59708	758	n/a	758(1.2)
Malaria*	59708	218*	n/a	218(0.3)

Residual risks (RRs) of TTIs

Our study comprised first-time donors only. Table 4 summarizes the IR and RR of TTIs in window period donations. After the implementation of NAT testing, the RR of HBV and HCV in our donors were 8.6 and 0.8 per million, respectively. These were 25.1 and 28.4 per million donors without NAT testing, respectively. This shows that NAT implementation significantly impacts residual risk reduction of HBV and HCV, which were 48.9% and 94.5%, respectively.

**Table 4:** Incidence and Residual Risk of Transfusion Transmitted Infections n/a=not available

Virus	Serological prevalence /10^5^ donation	Incident rate (per million donations)	Residual risk( per million donations)	Residual risk( per million donations) in the absence of NAT testing	% Risk Reduction
Hepatitis B	1486	14.05	8.6	25.1	48.9
Hepatitis C	1700	0.92	0.8	28.4	94.5
HIV	79	n/a	n/a	n/a	n/a

## Discussion

On a global level, the introduction of NAT in blood donor screening has significantly reduced the residual risk of TTIs. Residual risk estimates mainly depend upon the incidence rates of TTIs among blood donors, background prevalence of infection in the region, and the testing method used. Thus, it varies from country to country. This study showed that the residual risk of transmission of HCV has significantly dropped with NAT testing, i.e., 0.8 per million donors. However, it is still higher for HBV, i.e., 8.6 per million donors. The results are comparable to a local study by Moiz et al. in which the RR of HBV transmission was 1 in 10,900 donors, and for HCV, it was 1 in 13,900 donors [6]. These rates are relatively higher in India, as shown by Pandey et al. in a recently published study; they found RR with NAT of 16.1 per million donors and 4.4 per million donors for HBV and HCV, respectively [13]. Studies from western countries like US and Canada show that the RR of transmission of HCV and HIV is lower than that of HBV [14,15]. The plausible explanation is that HBV has a much higher tendency to cause occult blood infection than HCV; even extensive serological testing is not sufficient to detect HBV in its occult state.

NAT is the most sensitive diagnostic method suitable for donor screening. It has become mandatory to screen blood donations in developed countries [16-18]. However, NAT has been introduced for blood donor screening in Pakistan. Its high cost makes its availability limited to a few centers. Our study showed that overall cases detected by NAT are 34 out of 57759 samples tested, with 31 cases of HBV and only 3 cases of HCV. These results are comparable with other studies from Pakistan; Niazi et al. [7] showed 27 out of 54438 NAT reactive cases with 23 HBV reactive cases and 4 HCV reactive cases. Likewise, Awan et al. [9] showed 13 NAT reactive cases, 10 positives for HBV, and 3 donors positive for HCV. However, these findings contrast with another study from Pakistan, where Moiz et al. reported 27 HCV reactive cases with only 7 HBV reactive cases on NAT [6]. The prevalence of infection reported in all three studies is the same, with HCV being the most prevalent. The plausible explanation for high HBV yield is that HBsAg tests have certain limitations that have become a source of missing positive cases detected on NAT. These limitations include occult HBV infection (OBI) [19] and mutations in the major hydrophilic region of the HbsAg (the principal target for commercial HBsAg assays) [20]. Our results are also comparable with studies from Mediterranean countries where HCV is moderately endemic, but the NAT yield of HBV is higher than HCV. Stanic et al. reported 82 out of 545463 HBV NAT reactive cases, with only 16 HCV reactive cases [21]. A study from Slovenia reported the NAT yield of HBV infection as 1:15600 and the NAT yield of HCV infection as 1:524000 [22].

In contrast, HBV is the most prevalent TTI, with a high NAT yield in our neighboring countries, followed by HCV. A recent China study reported that NAT yields were 1062.90 per million and 0.97 per million for HBV and HCV, respectively [23]. Another study from India also showed 74 HBV, 12 HCV, and 1 HIV NAT positive cases in 1033,22 first-time donors tested [13]. A detailed comparison of NAT yield reported in different studies is shown in Table 5.

**Table 5:** Comparison of NAT yield with other studies NAT: Nucleic acid amplification test

Studies done	No. of donors tested	Incident cases	NAT yield	Yield rate
India [13]	103322	87	0.08	1 in 1187
India [24]	72794	121	0.16	1 in 601
China [23]	2,071,695	1763	0.08	1 in 1175
Turkey [25]	17792	13	0.06	1 in 1384
Pakistan [7]	54438	27	0.04	1 in 2016
Pakistan [6]	41304	49	0.12	1 in 845
This study	57759	34	0.05	1 in 1699

HIV has been on the rise since the detection of HIV in Pakistan in 1987 [26]. Multiple local HIV outbreaks have been reported so far [27]. By contrast prevalence of HIV along with NAT yield was negligible in our study, which is comparable to the other studies on blood donors from Pakistan [6,7,9]. This is credited to rigorous donor selection. However, the HIV NAT yield in neighboring countries is higher than in Pakistan. India reported an HIV NAT yield of 1 in 1250 donors [13], whereas China reported 1.45 per million donors [23]. Western countries like the US (0.43 per million) [10], Italy (1.8 per million), and Germany (0.43 per million) [28, 29] also have a high HIV prevalence and NAT yield. South Africa has reported the highest NAT yield for HIV(25.56 per million donations) among blood donors [30].

## Conclusions

NAT implementation has improved blood safety. Our study showed that NAT detected 34 out of 57759 cases initially missed by serological tests. This infected product could have become a source of infection for hundreds of people. We also found that NAT testing reduced the RR of HBV by 48.9% and HCV by 94.5%. The yield of NAT in detecting viral nucleic acid in blood was higher for HBV than for HCV. The study suggests that the parallel use of serology and NAT screening of donated blood would be beneficial.
